# Willingness to Access Peer-Delivered HIV Testing and Counseling Among People Who Inject Drugs in Bangkok, Thailand

**DOI:** 10.1007/s10900-012-9635-z

**Published:** 2012-11-13

**Authors:** Lianping Ti, Kanna Hayashi, Karyn Kaplan, Paisan Suwannawong, Evan Wood, Julio Montaner, Thomas Kerr

**Affiliations:** 1 Urban Health Research Initiative, British Columbia Centre for Excellence in HIV/AIDS, St. Paul’s Hospital, 608-1081 Burrard Street, Vancouver, BC V6Z 1Y6 Canada; 2 School of Population and Public Health, University of British Columbia, Vancouver, BC Canada; 3 Interdisciplinary Studies Graduate Program, University of British Columbia, Vancouver, BC Canada; 4 Thai AIDS Treatment Action Group, Bangkok, Thailand; 5 Department of Medicine, University of British Columbia, St. Paul’s Hospital, Vancouver, BC Canada

**Keywords:** Peer-delivered, Voluntary HIV counseling and testing, People who inject drugs, Thailand

## Abstract

Peer-based models for human immunodeficiency virus (HIV) testing have been implemented to increase access to testing in various settings. However, little is known about the acceptability of peer-delivered testing and counseling among people who inject drugs (IDU). During July and October 2011, data derived from the Mitsampan Community Research Project were used to construct three multivariate logistic regression models identifying factors associated with willingness to receive peer-delivered pre-test counseling, rapid HIV testing, and post-test counseling. Among a total of 348 IDU, 44, 38, and 36 % were willing to receive peer-delivered pre-test counseling, rapid HIV testing, and post-test counseling, respectively. In multivariate analyses, factors associated with willingness to access peer-delivered pre-test counseling included: male gender (adjusted odds ratio (AOR) = 0.48), higher than secondary education (AOR = 1.91), and binge drug use (AOR = 2.29) (all *p* < 0.05). Factors associated with willingness to access peer-delivered rapid HIV testing included: higher than secondary education (AOR = 2.06), binge drug use (AOR = 2.23), incarceration (AOR = 2.68), avoiding HIV testing (AOR = 0.24), and having been to the Mitsampan Harm Reduction Center (AOR = 1.63) (all *p* < 0.05). Lastly, binge drug use (AOR = 2.40), incarceration (AOR = 1.94), and avoiding HIV testing (AOR = 0.23) (all *p* < 0.05) were significantly associated with willingness to access peer-delivered post-test counseling. We found that a substantial proportion of Thai IDU were willing to receive peer-delivered HIV testing and counseling. These findings highlight the potential of peer-delivered testing to complement existing HIV testing programs that serve IDU.

## Introduction

Thailand continues to experience ongoing epidemics of illicit drug use and HIV infection among people who inject drugs (IDU). According to the Ministry of Public Health Thailand, the prevalence of HIV among this population remains high (between 30 and 50 %), while the prevalence of HIV in other high-risk groups, such as commercial sex workers and pregnant women, has been declining steadily over recent years [1]. To minimize the morbidity and mortality associated with HIV, many international health organizations are urging countries to scale up their voluntary HIV counseling and testing services (VCT) for IDU [2, 3], as testing can lead to the identification of undiagnosed HIV infection and early treatment [4–6]. In addition to linking IDU to proper healthcare services, knowledge of HIV serostatus may also have success in reducing HIV risk behavior among this population [7].

Although a large body of evidence supports increasing access to VCT in settings with high HIV epidemics among IDU [2, 8, 9], there exists an array of social and structural barriers that prevent IDU from accessing these services. Since the 2003 “War on Drugs” campaign launched by then Thai Prime Minster Thaksin Shinawatra, there has been continued reliance on drug law enforcement approaches to control drug trafficking and drug use in Thailand [10]. This has forced many IDU into hiding and has rendered them out of reach of potentially life-saving healthcare services [11]. Unfortunately, this policy approach has since been embraced by successive Thai governments despite lip service given to more public health-oriented approaches to dealing with illicit drug use and related harms [12–14]. Furthermore, stigmatizing attitudes of healthcare providers and the sharing of information between healthcare workers and police have been identified as factors that may cause some IDU to avoid conventional healthcare settings, including those that provide HIV testing services [11, 15]. Collectively, these social and structural barriers may contribute to a reluctance on the part of IDU to access VCT services, and consequently may increase their risk of HIV infection.

Peer-run services for IDU have been successful in extending the reach of traditional public health programs [16–18]. Task shifting, a term often used to describe the systematic delegation of tasks from physicians to workers with lower-level qualifications [19, 20], can be a strategy incorporated within peer-run services. In Africa, this decentralized approach has shown effectiveness in responding to the human health resource crisis [21, 22], especially during the current HIV/AIDS epidemic. Studies have demonstrated that with adequate training, lay workers were able to deliver healthcare services, including HIV testing [23], with comparable results to physicians [24]. In the context of IDU, expanding peer-run services to include task shifting from physicians to IDU may have potential to extend the reach of HIV services, and may minimize contact between IDU and healthcare workers who may hold stigmatizing attitudes toward the population [15].

While a large body of evidence supports task shifting as an approach to increasing access to VCT in various settings [22, 24], there are limited peer-based models of VCT for IDU in Thailand. Though peer-based models do exist in Thailand in the forms of peer-delivered education, prevention, support, and outreach activities [25, 26], peer-delivered HIV testing has yet to be explored in this context. Therefore, we sought to identify the prevalence and correlates of willingness to receive peer-delivered VCT among a community-recruited sample of IDU in Bangkok, Thailand.

## Methods

Data for these analyses were obtained from the Mitsampan Community Research Project, a collaborative research effort involving the Mitsampan Harm Reduction Center (MSHRC) (Bangkok, Thailand), the Thai AIDS Treatment Action Group (Bangkok, Thailand), Chulalongkorn University (Bangkok, Thailand), and the British Columbia Centre for Excellence in HIV/AIDS/University of British Columbia (Vancouver, Canada). During July and October 2011, the research partners undertook a cross-sectional study involving 440 community-recruited IDU. Participants were recruited through peer-based outreach efforts and word-of-mouth and were invited to attend the MSHRC or O-Zone House (drop-in centers for IDU in Bangkok operated by non-governmental organizations) to be part of the study. Individuals residing in Bangkok or in adjacent provinces who had injected drugs in the past 6 months were eligible for participation in the study. All participants provided informed consent and completed an interviewer-administered questionnaire eliciting a range of information, including demographic data, information on drug use patterns, HIV risk behavior, health problems, interactions with police and the criminal justice system, and experiences with healthcare. Upon completion of the questionnaire, participants received a stipend of 350 Thai Baht (approximately $12 USD). The study was approved by research ethics boards at Chulalongkorn University and the University of British Columbia.

For the present analyses, we restricted the study sample to individuals who were HIV-negative or of unknown HIV serostatus. The three dependent variables of interest included: (1) willingness to receive peer-delivered pre-test counseling; (2) willingness to receive peer-delivered rapid HIV testing; and (3) willingness to receive peer-delivered post-test counseling. These variables were ascertained by asking participants in a hypothetical scenario: “Who do you want to do your HIV pre-test counseling?”, “Who do you want to conduct/administer (do) the actual rapid HIV test?”, and “Who do you want to give you the result of the rapid HIV test (including post-test counseling)?”, respectively. Participants responded from the following options: doctor, nurse, trained peer, close friend, acquaintance, anyone, and other, and were allowed to check all that apply. Peer-delivery was defined as the receiving of HIV services by a trained former or current IDU. We compared IDU who were and were not willing to receive these services through peer-delivery using bivariate statistics and multivariate logistic regression. Variables considered included: median age (≥38 years old vs. <38 years old), gender (male vs. female), higher than secondary level education (≥secondary education vs. <secondary education), frequent heroin injection (>weekly vs. ≤weekly), frequent midazolam injection (>weekly vs. ≤weekly), injected with others on a frequent basis (>75 % vs. ≤75 %), binge drug use (yes vs. no), ever incarcerated (yes vs. no), ever avoid HIV testing (yes vs. no), ever experienced barriers to accessing healthcare services (any vs. none), had unprotected sex (yes vs. no), and ever been to MSHRC (yes vs. no). All behaviors and activities referred to the previous 6 months unless otherwise indicated. The barriers to accessing healthcare services variable included the following: limited hours of operation, long wait lists/times, didn’t know where to go, jail/detention/prison, no identification card (ID), my ID is registered somewhere else, no money, don’t want healthcare provider to know I use/inject drugs, was treated poorly by healthcare professionals, fear of sharing information of drug-using status with police, difficulty keeping appointments (e.g., have to miss work), transportation, or other. To examine bivariate associations, we used the Pearson χ^2^ test. Fisher’s exact test was used when one or more of the cells contained values less than or equal to five. As a next step, we applied an a priori-defined statistical protocol based on examination of the Akaike information criterion (AIC) and *p* values to construct three separate explanatory multivariate logistic regression models. First, we constructed each model by including all variables analyzed in bivariate analyses. After noting the AIC of the model, we removed the variable with the largest *p* value and built a reduced model. We continued this iterative process until no variables remained for inclusion. We selected the multivariate model with the lowest AIC score. All *p* values were two-sided.

## Results

In total, 348 IDU who were HIV-negative or of unknown HIV serostatus participated in this study; 68 (19.5 %) were female, and the median age was 38 years (IQR: 34–48 years). Among our study sample, 44, 38, and 36 % were willing to receive peer-delivered pre-test counseling, rapid HIV testing, and post-test counseling, respectively. As indicated in Table 1, in bivariate analyses, factors significantly associated with willingness to receive peer-delivered pre-test counseling included: male gender (odds ratio (OR) = 0.51; 95 % confidence interval (CI): 0.30–0.87), higher than secondary level education (OR = 2.01; 95 % CI: 1.29–3.14), midazolam injection more than once per week (OR = 1.71; 95 % CI: 1.12–2.63), injecting with others on a frequent basis (OR = 1.56; 95 % CI: 1.01–2.41), binge drug use (OR = 2.70; 95 % CI: 1.69–4.33), and having ever been to the MSHRC (OR = 1.64; 95 % CI: 1.07–2.51). Factors significantly associated with willingness to receive peer-delivered rapid HIV testing included: higher than secondary level education (OR = 1.75; 95 % CI: 1.11–2.75), binge drug use (OR = 2.24; 95 % CI: 1.40–3.58), incarceration (OR = 2.32; 95 % CI: 1.39–3.90), avoiding HIV testing (OR = 0.35; 95 % CI: 0.16–0.76), and having ever been to MSHRC (OR = 1.79; 95 % CI: 1.16–2.78). Lastly, peer-delivered post-test counseling was significantly and positively associated with binge drug use (OR = 2.24; 95 % CI: 1.40–3.59), incarceration (OR = 1.83; 95 % CI: 1.10–3.05), and negatively associated with avoiding HIV testing (OR = 0.28; 95 % CI: 0.12–0.64).

**Table 1:** Bivariate analyses of factors associated with willingness to receive peer-delivered pre-test counseling, rapid HIV testing, and post-test counseling among IDU in Bangkok, Thailand (n = 348)

Characteristic	Willingness to receive peer-delivered pre-test counseling		Willingness to receive peer-delivered rapid HIV-testing		Willingness to receive peer-delivered post-test counseling	
	Odds ratio (95 % CI)	*p* value	Odds ratio (95 % CI)	*p* value	Odds ratio (95 % CI)	*p* value
Age						
(≥38 years vs. <38 years)	0.86 (0.57–1.32)	0.50	1.09 (0.71–1.68)	0.70	1.04 (0.67–1.61)	0.85
Gender						
(Male vs. female)	0.51 (0.30–0.87)	0.01	0.73 (0.42–1.24)	0.24	0.77 (0.45–1.32)	0.34
Education						
(≥Secondary education vs. <secondary education)	2.01 (1.29–3.14)	<0.01	1.75 (1.11–2.75)	0.02	1.55 (0.98–2.45)	0.06
Heroin injection*						
(>Weekly vs. ≤weekly)	1.63 (0.95–2.80)	0.08	1.20 (0.70–2.09)	0.51	0.88 (0.50–1.55)	0.66
Midazolam injection*						
(>Weekly vs. ≤weekly)	1.71 (1.12–2.63)	0.01	1.18 (0.76–1.82)	0.46	1.13 (0.73–1.75)	0.58
Inject with others*						
(>75 % vs. ≤75 %)	1.56 (1.01–2.41)	0.05	1.29 (0.83–2.01)	0.26	1.12 (0.72–1.76)	0.61
Binge drug use*						
(Yes vs. no)	2.70 (1.69–4.33)	<0.01	2.24 (1.40–3.58)	<0.01	2.24 (1.40–3.59)	<0.01
Ever incarcerated						
(Yes vs. no)	1.08 (0.68–1.73)	0.74	2.32 (1.39–3.90)	<0.01	1.83 (1.10–3.05)	0.02
Avoid HIV testing						
(Yes vs. no)	0.58 (0.30–1.11)	0.10	0.35 (0.16–0.76)	<0.01	0.28 (0.12–0.64)	<0.01
Barriers to accessing healthcare services						
(Any vs. none)	1.30 (0.81–2.09)	0.28	0.86 (0.54–1.39)	0.55	0.73 (0.45–1.18)	0.20
Unprotected sex*						
(Yes vs. no)	0.69 (0.44–1.08)	0.10	0.79 (0.50–1.24)	0.30	0.79 (0.50–1.25)	0.31
Ever been to MSHRC						
(Yes vs. no)	1.64 (1.07–2.51)	0.02	1.79 (1.16–2.78)	<0.01	1.46 (0.94–2.26)	0.09

*IDU* people who inject drugs, *CI* confidence interval, *MSHRC* Mitsampan Harm Reduction Center

* Refers to behavior/activities in the previous 6 months

As indicated in Table 2, in multivariate analyses, factors that remained significantly associated with willingness to receive peer-delivered pre-test counseling were male gender (adjusted odds ratio (AOR) = 0.48; 95 % CI; 0.27–0.85), higher than secondary level education (AOR = 1.91; 95 % CI: 1.20–3.06), and binge drug use (AOR = 2.29; 95 % CI: 1.40–3.77). Higher than secondary level education (AOR = 2.06; 95 % CI: 1.27–3.39), binge drug use (AOR = 2.23; 95 % CI: 1.36–3.70), incarceration (AOR = 2.68; 95 % CI: 1.56–4.72), avoiding HIV testing (AOR = 0.24; 95 % CI: 0.10–0.52), and having ever been to the MSHRC (AOR = 1.63; 95 % CI: 1.02–2.62) remained significantly associated with willingness to receive peer-delivered rapid HIV testing. Lastly, factors that were significantly and independently associated with willingness to receive peer-delivered post-test counseling included: binge drug use (AOR = 2.40; 95 % CI: 1.48–3.93), incarceration (AOR = 1.94; 95 % CI: 1.16–3.33), and avoiding HIV testing (AOR = 0.23; 95 % CI: 0.09–0.52).

**Table 2:** Multivariate logistic regression analyses of factors associated with willingness to receive peer-delivered pre-test counseling, rapid HIV testing, and post-test counseling among IDU in Bangkok, Thailand (n = 348)

Characteristic	Willingness to receive peer-delivered pre-test counseling		Willingness to receive peer-delivered rapid HIV-testing		Willingness to receive peer-delivered post-test counseling	
	AOR (95 % CI)	*p* value	AOR (95 % CI)	*p* value	AOR (95 % CI)	*p* value
Gender						
(Male vs. female)	0.48 (0.27–0.85)	0.01	–	–	–	–
Education						
(≥Secondary education vs. <secondary education)	1.91 (1.20–3.06)	<0.01	2.06 (1.27–3.39)	<0.01	–	–
Midazolam injection*						
(>Weekly vs. ≤weekly)	1.46 (0.92–2.32)	0.11	–	–	–	–
Binge drug use*						
(Yes vs. no)	2.29 (1.40–3.77)	<0.01	2.23 (1.36–3.70)	<0.01	2.40 (1.48–3.93)	<0.01
Ever incarcerated						
(Yes vs. no)	–	–	2.68 (1.56–4.72)	<0.01	1.94 (1.16–3.33)	0.01
Avoid HIV testing						
(Yes vs. no)	–	–	0.24 (0.10–0.52)	<0.01	0.23 (0.09–0.52)	<0.01
Ever been to MSHRC						
(Yes vs. no)	1.43 (0.90–2.26)	0.13	1.63 (1.02–2.62)	0.04	–	–

*IDU* people who inject drugs, *CI* confidence interval, *AOR* adjusted odds ratio, *MSHRC* Mitsampan Harm Reduction Center

* Refers to behavior/activities in the previous 6 months

## Discussion

In the present study, we found that a considerable proportion of Thai IDU were willing to receive peer-delivered VCT, with 44, 38, and 36 % of the study participants willing to receive peer-delivered pre-test counseling, rapid HIV testing, and post-test counseling, respectively. In multivariate analyses, willingness to receive peer-delivered pre-test counseling was significantly and positively associated with binge use and having higher than secondary level education, and negatively associated with male gender. Multivariate results also concluded that willingness to receive peer-delivered rapid HIV testing was positively associated with having higher than secondary level education, binge use, a history of incarceration, and having previously been to MSHRC, while negatively associated with avoiding HIV testing. Lastly, factors positively associated with willingness to receive peer-delivered post-test counseling were binge use and incarceration, whereas avoiding HIV testing was negatively associated with the dependent variable.

Our findings reveal that many IDU in our study were willing to receive VCT through peer-delivery. This supports previous studies conducted in Sub-Saharan Africa that have demonstrated the potential of employing lay healthcare workers to deliver HIV services [22, 24, 27]. Furthermore, these results complement a recent study conducted in Bangkok [28]. which indicated that approximately three-quarters of Thai IDU were willing to receive HIV testing at a drug user-run drop-in center. Although over a third of the participants were willing to access peer-delivered VCT, the rate of willingness observed here may reflect the fact that many IDU avoid HIV testing altogether and many are already being tested regularly through other sources and therefore likely do not see a need to get tested elsewhere [29, 30]. The finding that willingness to receive peer-delivered testing and post-test counseling drops by 6 % compared to pre-test counseling may be due to concerns over patient confidentiality among peers as well as the perceived ability of peers to administer HIV tests [31]. Future research using in-depth qualitative research methods are needed to understand the reasons why Thai IDU would be willing or unwilling to access peer-delivered VCT services. Nevertheless, given the various barriers that Thai IDU may face in accessing HIV services, such as HIV-related stigma and discrimination within healthcare settings [15], peer-delivered interventions that complement existing HIV programs may have potential to increase the uptake of HIV testing among this population.

The results from our study showed that binge drug use was strongly associated with all three outcomes: willingness to receive peer-delivered rapid HIV testing and pre- and post-test counseling. That IDU engaging in high intensity drug use were more willing to access these services is encouraging, given that these individuals are at higher risk of transmitting HIV through syringe sharing [32–34]. The fact that these individuals were twice as likely to receive peer-delivered HIV services than less heavy users could again be due to the stigmatization and discrimination by healthcare workers associated with being a high intensity drug user [15]. In light of these findings, efforts to implement peer-delivered VCT in settings outside of conventional healthcare settings may be important for reaching these individuals at heightened risk of HIV.

We further observed that factors associated with peer-delivered HIV testing and post-test counseling were very similar, meaning that IDU who were willing to receive peer-delivered HIV testing were also willing to receive post-test counseling through peer-delivery. This may reflect the fact that these two services complement each other, since post-test counseling refers to the process in which patients receive the results of their HIV test, discuss harm reduction strategies, and receive referrals to clinics or hospitals for further care and support [3, 35]. Our study found that having a history of incarceration was positively associated with willingness to receive both peer-delivered rapid HIV testing and post-test counseling. Given that some hospitals collect and share information concerning suspecting drug users with police [11], and that previously incarcerated IDU may fear future confrontations with police and re-incarceration, this finding may reflect the fact that these individuals may be more reluctant to access services provided in conventional public health settings. Given the large body of evidence indicating an elevated risk of HIV transmission among incarcerated IDU [36–38], it is reassuring that these individuals were more likely to access peer-delivered VCT services outside of conventional clinical environments.

Our findings also indicated that IDU who reported avoiding HIV testing were less likely to receive peer-delivered rapid HIV testing and post-test counseling. This negative association may suggest that these individuals are avoiding testing altogether, regardless of where the testing is taking place (i.e., conventional healthcare settings or peer-run drop-in centers). Various reasons for avoiding testing can include: fear of an HIV-positive test result, fear of negative reactions from family and the community, and feelings of shame and hopelessness [39–41].

Our study also found that IDU with lower than secondary level education were less likely to access peer-delivered pre-test counseling and rapid HIV testing compared to those with higher level education. These individuals may not be aware of the benefits of HIV testing and may lack knowledge of the risks associated with HIV transmission [28, 42]. In light of this finding, increased efforts for targeted outreach, educational HIV prevention interventions, and information on HIV care and treatment support are needed to reach these individuals.

Our finding that participants who have previously been to the MSHRC were more likely to get peer-delivered HIV testing suggests that drug user-run drop-in centers may serve as an additional setting for the delivery of healthcare services to Thai IDU. This supports a large body of evidence which demonstrates the value of peer-run interventions in supporting and increasing access to public health programs [16, 43, 44]. While offering services at a peer-run harm reduction center may be effective in attracting a larger number of IDU, a study by Kerr and colleagues found that female IDU were less likely to access the MSHRC [18]; yet the findings from the present study indicate that female IDU may be more willing to access peer-delivered pre-test counseling. Thus, efforts should be made to increase awareness of and access to the MSHRC, especially among these individuals.

This study has several limitations. First, due to the cross-sectional design of the study, we were unable to determine a temporal relationship between the explanatory variables and our three outcomes of interest. Second, the data collected were self-reported and may be subject to reporting biases, such as socially desirable reporting and recall biases. Third, since the study sample was not randomly selected, the study findings may not be representative of Thai IDU. Hence, this study may not be generalizable to Thai IDU and IDU in other settings.

In sum, a substantial proportion of Thai IDU were willing to receive peer-delivered VCT if it were offered at the MSHRC or similar peer-run drop-in centers. Further, our study revealed that these individuals were more likely to be engaged in high intensity drug use and have been previously incarcerated. These findings provide evidence supporting the implementation and evaluation of novel approaches to HIV testing for IDU to complement existing programs offered in conventional healthcare settings.
